# State-of-the-art of artificial intelligence in medicinal chemistry

**DOI:** 10.2144/fsoa-2021-0030

**Published:** 2021-03-29

**Authors:** Jürgen Bajorath

**Affiliations:** 1 Department of Life Science Informatics and Data Science, B-IT, LIMES Program Unit Chemical Biology & Medicinal Chemistry, Rheinische Friedrich-Wilhelms-Universität, Friedrich-Hirzebruch-Allee 6, Bonn D, 53115, Germany

**Keywords:** artificial intelligence, big data, chemical reaction modeling, compound design, machine learning, medicinal chemistry, property prediction, small data

As in many areas of science and technology, artificial intelligence (AI) is currently promoted with high expectations in drug discovery and medicinal chemistry. Here, AI mostly refers to machine learning (ML), which is only a part of the methodological AI spectrum. The high level of interest in AI essentially originates from deep learning using multi-layered neural network (NN) architectures. Other AI approaches entering medicinal chemistry include expert systems and (laboratory) robotics. However, deep learning is clearly predominant. Of note, ML already has a long history in chemoinformatics and medicinal chemistry. For more than two decades, ML methods have been extensively applied for compound property predictions. In medicinal chemistry, properties of interest for computational studies include, first and foremost, biological activities of small molecules, but also physiochemical (e.g., solubility) or *in vivo* properties (such as metabolic stability or toxicity). Predictions of such properties aim to support the key task in the practice of medicinal chemistry: deciding which compound(s) to synthesize next. Over the years, NNs – which were popular early on for property predictions – were for the most part replaced by other ML methods such as support vector machines, random forests or Bayesian modeling. This was largely due to the tendency of NNs to overfit models to training data and also to the black box character of their predictions (the black box also applies to other – but not all – ML methods). In medicinal chemistry, chemical intuition continues to play a major role and black box predictions that cannot be explained in chemical terms work against the acceptance of ML for practical applications. Recently, NNs have been experiencing a renaissance in medicinal chemistry with the advent of deep neural networks (DNNs) and high expectations associated with deep ML. These expectations have primarily originated from other fields such as computer vision (image analysis), natural language processing or network science (including social networks).

## Balancing high hopes

In a publication comparing black box models, explainable and interpretable ML from a computer science perspective [1], Cynthia Rudin states the following: *“When considering problems that have structured data with meaningful features, there is often no significant difference in performance between more complex classifiers (deep neural networks, boosted decision trees, random forests) and much simpler classifiers (logistic regression, decision lists) after preprocessing.”* This situation exactly applies to medicinal chemistry. Here, one typically does not work with very large sets of low-resolution data (such a pixels comprising images) and apply representation learning, a notable strength of DNNs. Instead, in medicinal chemistry projects, available data sets are mostly small and (mathematically) defined molecular representations are used (e.g., molecular graph-based descriptor functions). Such conditions explain observations that are frequently made: at least for property prediction in medicinal chemistry, there is little, if any detectable advantage of DNNs over other (simpler) ML methods.

In her article, Cynthia Rudin makes another key point that is equally applicable to medicinal chemistry and drug design: *“There is a widespread belief that more complex models are more accurate, meaning that a complicated black box is necessary for top predictive performance. However, this is often not true …”* [1]. One may rephrase this point and emphasize that methodological complexity does not necessarily scale with predictive performance. Clearly, no complex ML approach is ‘validated’ until it is not conclusively demonstrated that simpler methods do not yield comparable results; a critical issue that is often not sufficiently considered in the literature.

## State-of-the-art

While property predictions in medicinal chemistry benefit only little from deep learning, for the reasons discussed, DNNs open the door to novel types of applications, for example, in generative molecular design [2] or chemical reaction analysis [3]. This is a scientifically stimulating aspect of the current AI ecstasy.

Generative compound design aims to construct chemically novel molecules with desired properties. It is also applied to generate ultra-large virtual libraries. For generative compound design or reaction modeling, specific DNN architectures have been adapted and further evolved. As an example, recurrent NNs represent a versatile computational architecture that consists of an encoder-decoder framework with integral latent design space. The framework is composed of long short-term memory units. The internal memory enables recurrent NNs to learn from sequential data, yielding so-called sequence-to-sequence models for transforming one data (string) sequence into another.

Recent applications of this and other DNN architectures illustrate scientific heterogeneity of the field. In the view of the author, substantial progress has been made in synthesis design and the discovery or classification of chemical reactions (see, e.g., [3–5]). However, in generative modeling, *de novo* design and compound repurposing, significant advances are less evident. Despite reports of individual success stories (e.g., [6]), caveats are along the way [7] and successful parallel applications of a given approach on different compound classes are currently lacking. While elegant compound design strategies can be implemented using DNN architectures (and have also been studied in our laboratory, e.g., [8]), the jury is still out whether or not DNNs are capable of generating new chemical entities that are of higher quality than others or represent an unusually high degree of chemical novelty. While there are recurrent claims by drug discovery start-ups operating under the AI label of breakthroughs in generating novel proprietary compounds in record times, such claims cannot be assessed scientifically and substantiated if the data are not made available. Similar claims are also found in peer-reviewed publications that are often difficult to support scientifically. Whether or not generative *de novo* design might help to increase the success rate of hit-to-lead and lead optimization programs in medicinal chemistry essentially remains an open question at present.

In general, reports of practical AI applications with a clear positive impact on medicinal chemistry are still rare [9]. Moreover, currently available studies also indicate that candidate compounds from ML continue to be prioritized by domain experts, rather than machines. From this point of view, we are still far away from ‘true’ AI in discovery settings where compound decisions would be made by algorithms beyond human reasoning [9].

## Big data versus small data

Benefits of DNNs are often associated with learning from ‘big data’ (characteristic features go beyond mere data volumes) [10]. In recent years, big data trends are also witnessed in medicinal chemistry [10]. In February 2021, major public repositories of compound data (ChEMBL, PubChem, ZINC) contained more than 2 million bioactive compounds from the medicinal chemistry literature with 16 million activity annotations for more than 13,000 biological targets. In addition, more than 1.2 million biological assays with 273 million activity data points were available. Furthermore, more than 200 million commercial compounds were offered for medicinal chemistry. Of course, for investigators working in particle physics, genomics or social media, such data volumes might be a far cry from what they perceive as big data. However, for a field such as medicinal chemistry that is traditionally not data-driven, these volumes are challenging. Learning from such compound and activity data through large-scale data analytics provides new opportunities for the further development of medicinal chemistry as a scientific discipline [11]. Importantly, the situation is different for predictive modeling. In medicinal chemistry, ML is mostly applied at the level of individual (target-directed) projects and hence based upon ‘small data’, as discussed above. In data science, the context dependence of data structuring and analysis is known to work against generalization of knowledge extraction, which requires abstraction from project-based data sets and project-specific analysis criteria [12]. By contrast, in medicinal chemistry, project focus takes center stage and limits data and information available for ML.

The predominant small data context in medicinal chemistry also suggests alternative strategies for ML. Rather than heavily investigating methodologies whose strengths depend upon large data volumes and representation learning, it is meaningful to consider approaches that are capable of predicting properties or new compounds on the basis of sparse data such as transfer or active learning. Transfer learning makes it possible to use data from related prediction tasks (targets) for modeling; active learning derives predictive models from minimal sets of most informative training instances. In medicinal chemistry, this situation particularly applies to novel targets with interesting disease biology for which much less compound information is available than for popular therapeutic targets that have already been heavily investigated.

## Conclusion

AI is increasingly considered in medicinal chemistry such as in many other scientific fields. Notably, ML already has a long history in medicinal chemistry, in particular, for compound property prediction. While big data trends are detectable in medicinal chemistry, ML is mostly applied in the context of project-specific small data regimes using well-defined molecular representations. Under these conditions, DNNs offer essentially no advantages over other ML approaches. However, the use of DNN architectures enables new types of applications that would be difficult to tackle otherwise. In some areas such as chemical reaction modeling, synthesis design or laboratory robotics, notable progress is being made. In others such as generative modeling for *de novo* compound design, heralded advances are less evident (and partly oversold). While the AI label is currently used to promote many computational activities in the field (which frequently are far away from AI), practical (prospective) applications of deep ML with a demonstrated impact on medicinal chemistry projects are still rare. Moreover, most studies computing new compounds continue to rely on expert knowledge at the candidate selection stage. All in all, there is much room for further developments with demonstrated practical utility. Clearly, ML and DNNs are here to stay in medicinal chemistry, but claims and expectations should be balanced to further improve credibility and acceptance in the field. It will be exciting to explore new applications that exploit strengths of AI. Moreover, considering the prevalent small data context of medicinal chemistry projects, especially for novel targets, another stimulating task will be focusing on computational approaches that can generate sound predictive models on the basis of minimal learning sets.

## References

[1] Rudin C. Stop explaining black box machine learning models for high stakes decisions and use interpretable models instead. Nat. Mach. Intell. 1(5), 206–215 (2019). 10.1038/s42256-019-0048-x PMC9122117 35603010

[2] Struble TJ, Alvarez JC, Brown SP Current and future roles of artificial intelligence in medicinal chemistry synthesis. J. Med. Chem. 63(16), 8667–8682 (2020). 32243158 10.1021/acs.jmedchem.9b02120 PMC7457232

[3] Chen H, Engkvist O, Wang Y, Olivecrona M, Blaschke T. The rise of deep learning in drug discovery. Drug Discov. Today 23(6), 1241–1250 (2018). 29366762 10.1016/j.drudis.2018.01.039

[4] Bort W, Baskin II, Gimadiev T Discovery of novel chemical reactions by deep generative recurrent neural network. Sci. Rep. 11(1), 1–15 (2021). 33542271 10.1038/s41598-021-81889-y PMC7862614

[5] Schwaller P, Probst D, Vaucher AC Mapping the space of chemical reactions using attention-based neural networks. Nat. Mach. Intell. (2021). 10.1038/s42256-020-00284-w

[6] Stokes JM, Yang K, Swanson K A deep learning approach to antibiotic discovery. Cell 180(4), 688–702 (2020). 32084340 10.1016/j.cell.2020.01.021 PMC8349178

[7] Walters WP, Murcko M. Assessing the impact of generative AI on medicinal chemistry. Nat. Biotechnol. 38(2), 143–145 (2020). 32001834 10.1038/s41587-020-0418-2

[8] Yoshimori A, Bajorath J. Deep SAR matrix: SAR matrix expansion for advanced analog design using deep learning architectures. Future Drug Discov. 2(2), FDD36 (2020).

[9] Bajorath J, Kearnes S, Walters WP, Meanwell NA, Georg GI, Wang S. Artificial intelligence in drug discovery: into the great wide open. J. Med. Chem. 63(16), 8651–8652 (2020). 32639156 10.1021/acs.jmedchem.0c01077

[10] Hu Y, Bajorath J. Entering the ‘big data’ era in medicinal chemistry: molecular promiscuity analysis revisited. Future Sci. OA 3(2), FSO179 (2017). 28670471 10.4155/fsoa-2017-0001 PMC5481856

[11] Bajorath J. Foundations of data-driven medicinal chemistry. Future Sci. OA 4(8), FSO320 (2018). 30271612 10.4155/fsoa-2018-0057 PMC6153455

[12] Provost F, Fawcett T. Data science and its relationship to big data and data-driven decision making. Big Data 1(1), 51–59 (2013). 27447038 10.1089/big.2013.1508

